# 4-(4-Methoxy­phen­yl)piperazin-1-ium chloride

**DOI:** 10.1107/S1600536809004280

**Published:** 2009-02-11

**Authors:** Zia-ur-Rehman, M. Nawaz Tahir, Muhammad Danish, Niaz Muhammad, Saqib Ali

**Affiliations:** a Department of Chemistry, Quaid-i-Azam University, Islamabad 45320, Pakistan; b University of Sargodha, Department of Physics, Sargodha, Pakistan; c University of Sargodha, Department of Chemistry, Sargodha, Pakistan

## Abstract

In the title compound, C_11_H_17_N_2_O^+^·Cl^−^, the dihedral angle between the benzene ring and the basal plane of piperazine ring is 39.20 (8)°. In the crystal, intermolecular N—H⋯Cl hydrogen bonds occur.  There is also a C—H⋯π inter­action between the benzene rings.

## Related literature

The title compound was obtained as a by-product in a contin­uation of work on the synthesis of tin complexes containing piperazine, see: Zia-ur-Rahman *et al.* (2006 ▶, 2007 ▶). For related structures, see: Lu (2007 ▶); Sadiq-ur-Rehman *et al.* (2007 ▶).
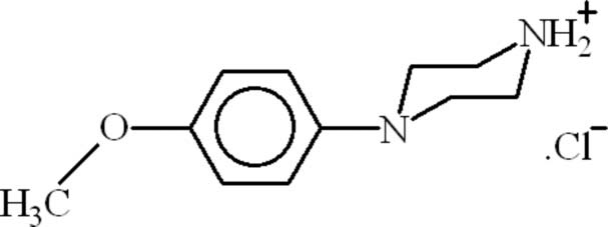


## Experimental

### 

#### Crystal data

C_11_H_17_N_2_O^+^·Cl^−^
*M* *_r_* = 228.72
Orthorhombic, 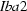

*a* = 10.2890 (7) Å
*b* = 31.5218 (18) Å
*c* = 7.5909 (5) Å
*V* = 2461.9 (3) Å^3^
*Z* = 8
Mo *K*α radiation
μ = 0.29 mm^−1^
*T* = 296 (2) K
0.28 × 0.22 × 0.15 mm

#### Data collection

Bruker KAPPA APEXII CCD diffractometer
Absorption correction: multi-scan (*SADABS*; Bruker, 2005 ▶) *T*_min_ = 0.922, *T*_max_ = 0.950
7605 measured reflections
3073 independent reflections
2327 reflections with *I* > 2σ(*I*)
*R*_int_ = 0.026

#### Refinement

*R*[*F* ^2^ > 2σ(*F* ^2^)] = 0.044
*wR*(*F* ^2^) = 0.139
*S* = 1.01
3073 reflections
137 parameters
1 restraint
H-atom parameters constrained
Δρ_max_ = 0.35 e Å^−3^
Δρ_min_ = −0.29 e Å^−3^
Absolute structure: Flack (1983 ▶), 1076 Friedel pairs
Flack parameter: 0.05 (9)

### 

Data collection: *APEX2* (Bruker, 2007 ▶); cell refinement: *SAINT* (Bruker, 2007 ▶); data reduction: *SAINT*; program(s) used to solve structure: *SHELXS97* (Sheldrick, 2008 ▶); program(s) used to refine structure: *SHELXL97* (Sheldrick, 2008 ▶); molecular graphics: *ORTEP-3 for Windows* (Farrugia, 1997 ▶) and *PLATON* (Spek, 2009 ▶); software used to prepare material for publication: *WinGX* publication routines (Farrugia, 1999 ▶) and *PLATON*.

## Supplementary Material

Crystal structure: contains datablocks global, I. DOI: 10.1107/S1600536809004280/at2720sup1.cif

Structure factors: contains datablocks I. DOI: 10.1107/S1600536809004280/at2720Isup2.hkl

Additional supplementary materials:  crystallographic information; 3D view; checkCIF report

## Figures and Tables

**Table 1:** Hydrogen-bond geometry (Å, °)

*D*—H⋯*A*	*D*—H	H⋯*A*	*D*⋯*A*	*D*—H⋯*A*
N2—H2*A*⋯Cl1^i^	0.90	2.20	3.082 (2)	168
N2—H2*B*⋯Cl1	0.90	2.24	3.134 (3)	177
C3—H3⋯CgA^ii^	0.93	2.88	3.573 (2)	133

Symmetry codes: (i) 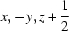; (ii) 
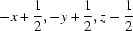
. *CgA* is the centroid of the benzene ring.

**Fig. 1.**
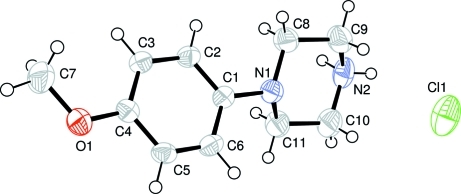
ORTEP drawing of the title compound, with the atom numbering scheme. The thermal ellipsoids are drawn at the 50% probability level. H-atoms are shown by small circles of arbitrary radii.

**Fig. 2.**
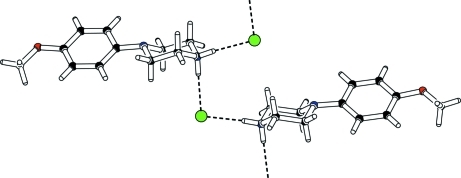
The partial packing figure (PLATON: Spek, 2009) which shows the dimeric nature of the compound.

## supplementary crystallographic information

### Comment

In continuation to synthesizing the tin complexes containing piperazine (Zia-ur-Rahman *et al.*, 2006, 2007), the title compound (I), (Fig 1) has been obtained as a byproduct.

The crystal structures of (II) 4-nitrophenylpiperazinium chloride monohydrate (Lu, 2007) and 4-(2-pyridyl)piperazin-1-ium chloride (Sadiq-ur-Rehman *et al.*, 2007) has been reported. The title compound have a replacement of nitro group in (II) with methoxy at the same position. Due to this change it is observed that (I) does not contain water molecule although the aquas medium was present during crystallization. In the title compound the benzene ring A(C1—C6) is planar along with the methoxy group. The piperazinium is in chair form with the basal plane B(C8—C11) and the N–atoms are at a distance of 0.6680 (37) and -0.6620 (46) Å. The dihedral angle between the groups A and B is 39.20 (8)°. The molecules are linked each other through intra and intermolecular H–bonding (Table 1, Fig 2). There exists a π interaction between the C3–H3 and the centroid of the benzene ring.

### Experimental

Me_2_SnCl_2_ (0.24 g, 1.08 mmol) in methanol (30 ml) was added dropwise to 4-(4-methoxyphenyl)piperazinium 4-(4-methoxyphenyl)piperazine-1-carbodithioate (0.5 g, 1.08 mmol) in methanol (30 ml) and the mixture was refluxed for 3 h with constant stirring. The 4-(4-methoxyphenyl)piperazinium chloride thus formed, was filtered off and recrystallized from water-ethanol (1:4) to give colourless crystals.

### Refinement

All H atoms were positioned geometrically and refined using a riding model, with N–H = 0.90Å and with *U*_iso_(H) = 1.2*U*_eq_(C), and with C–H = 0.93–0.97Å and with *U*_iso_(H) = 1.2*U*_eq_(C) [C–H = 0.96Å and *U*_iso_(H) = 1.5*U*_eq_(C) for the methyl group].

### Crystal data

**Table:**

C_11_H_17_N_2_O^+^·Cl^−^	*F*(000) = 976
*M**_r_* = 228.72	*D*_x_ = 1.234 Mg m^−^^3^
Orthorhombic, *I**b**a*2	Mo *K*α radiation, λ = 0.71073 Å
Hall symbol: I 2 -2c	Cell parameters from 3073 reflections
*a* = 10.2890 (7) Å	θ = 1.2–30.5°
*b* = 31.5218 (18) Å	µ = 0.29 mm^−^^1^
*c* = 7.5909 (5) Å	*T* = 296 K
*V* = 2461.9 (3) Å^3^	Prismatic, colourless
*Z* = 8	0.28 × 0.22 × 0.15 mm

### Data collection

**Table:**

Bruker KAPPA APEXII CCD diffractometer	3073 independent reflections
Radiation source: fine-focus sealed tube	2327 reflections with *I* > 2σ(*I*)
graphite	*R*_int_ = 0.026
Detector resolution: 7.3 pixels mm^-1^	θ_max_ = 30.5°, θ_min_ = 1.3°
ω scans	*h* = −14→12
Absorption correction: multi-scan (*SADABS*; Bruker, 2005)	*k* = −42→44
*T*_min_ = 0.922, *T*_max_ = 0.950	*l* = −5→10
7605 measured reflections	

### Refinement

**Table:**

Refinement on *F*^2^	Hydrogen site location: inferred from neighbouring sites
Least-squares matrix: full	H-atom parameters constrained
*R*[*F*^2^ > 2σ(*F*^2^)] = 0.044	*w* = 1/[σ^2^(*F*_o_^2^) + (0.0883*P*)^2^ + 0.0737*P*] where *P* = (*F*_o_^2^ + 2*F*_c_^2^)/3
*wR*(*F*^2^) = 0.139	(Δ/σ)_max_ < 0.001
*S* = 1.00	Δρ_max_ = 0.35 e Å^−^^3^
3073 reflections	Δρ_min_ = −0.29 e Å^−^^3^
137 parameters	Extinction correction: *SHELXL*, Fc^*^=kFc[1+0.001xFc^2^λ^3^/sin(2θ)]^-1/4^
1 restraint	Extinction coefficient: 0.0136 (13)
Primary atom site location: structure-invariant direct methods	Absolute structure: Flack (1983), 1076 Friedel pairs
Secondary atom site location: difference Fourier map	Flack parameter: 0.05 (9)

### Special details

**Table:**

Geometry. Bond distances, angles *etc*. have been calculated using the rounded fractional coordinates. All su's are estimated from the variances of the (full) variance-covariance matrix. The cell e.s.d.'s are taken into account in the estimation of distances, angles and torsion angles
Refinement. Refinement of *F*^2^ against ALL reflections. The weighted *R*-factor *wR* and goodness of fit *S* are based on *F*^2^, conventional *R*-factors *R* are based on *F*, with *F* set to zero for negative *F*^2^. The threshold expression of *F*^2^ > σ(*F*^2^) is used only for calculating *R*-factors(gt) *etc*. and is not relevant to the choice of reflections for refinement. *R*-factors based on *F*^2^ are statistically about twice as large as those based on *F*, and *R*- factors based on ALL data will be even larger.

### Fractional atomic coordinates and isotropic or equivalent isotropic displacement parameters (Å^2^)

**Table:**

	*x*	*y*	*z*	*U*_iso_*/*U*_eq_	
O1	0.41690 (13)	0.30736 (4)	0.7357 (3)	0.0454 (4)	
N1	0.30676 (15)	0.13295 (5)	0.7320 (3)	0.0407 (5)	
N2	0.25384 (18)	0.04631 (6)	0.6512 (4)	0.0561 (8)	
C1	0.33328 (16)	0.17757 (5)	0.7214 (3)	0.0334 (5)	
C2	0.25085 (17)	0.20603 (6)	0.6364 (3)	0.0368 (6)	
C3	0.27578 (17)	0.24960 (7)	0.6389 (3)	0.0362 (6)	
C4	0.38456 (16)	0.26505 (6)	0.7235 (3)	0.0329 (5)	
C5	0.46885 (18)	0.23669 (6)	0.8075 (3)	0.0371 (5)	
C6	0.44271 (18)	0.19397 (6)	0.8060 (3)	0.0386 (6)	
C7	0.3309 (3)	0.33675 (8)	0.6540 (4)	0.0641 (9)	
C8	0.1772 (2)	0.11964 (7)	0.6751 (3)	0.0486 (7)	
C9	0.1533 (2)	0.07386 (7)	0.7291 (4)	0.0563 (7)	
C10	0.3855 (3)	0.06000 (7)	0.7054 (4)	0.0617 (9)	
C11	0.4071 (2)	0.10588 (7)	0.6540 (4)	0.0513 (7)	
Cl1	0.24567 (7)	0.04913 (2)	0.23863 (13)	0.0710 (3)	
H2	0.17815	0.19587	0.57701	0.0442*	
H2A	0.24059	0.01935	0.68585	0.0673*	
H2B	0.24770	0.04718	0.53299	0.0673*	
H3	0.21888	0.26823	0.58342	0.0435*	
H5	0.54265	0.24678	0.86451	0.0446*	
H6	0.49937	0.17549	0.86277	0.0464*	
H7A	0.36281	0.36507	0.67113	0.0962*	
H7B	0.32577	0.33080	0.53014	0.0962*	
H7C	0.24605	0.33421	0.70556	0.0962*	
H8A	0.11208	0.13783	0.72828	0.0583*	
H8B	0.17019	0.12226	0.54811	0.0583*	
H9A	0.06804	0.06493	0.68906	0.0675*	
H9B	0.15557	0.07151	0.85649	0.0675*	
H10A	0.39486	0.05691	0.83195	0.0742*	
H10B	0.45021	0.04222	0.64893	0.0742*	
H11A	0.40474	0.10852	0.52670	0.0615*	
H11B	0.49212	0.11503	0.69420	0.0615*	

### Atomic displacement parameters (Å^2^)

**Table:**

	*U*^11^	*U*^22^	*U*^33^	*U*^12^	*U*^13^	*U*^23^
O1	0.0468 (7)	0.0384 (7)	0.0510 (9)	−0.0060 (5)	−0.0035 (8)	0.0000 (8)
N1	0.0415 (8)	0.0359 (7)	0.0447 (9)	0.0009 (6)	0.0001 (9)	0.0004 (8)
N2	0.0814 (15)	0.0327 (10)	0.0542 (14)	−0.0018 (8)	−0.0042 (10)	0.0048 (9)
C1	0.0351 (8)	0.0346 (8)	0.0306 (9)	0.0019 (6)	0.0006 (9)	−0.0009 (8)
C2	0.0353 (9)	0.0401 (10)	0.0351 (11)	−0.0007 (7)	−0.0065 (8)	0.0002 (8)
C3	0.0345 (9)	0.0382 (10)	0.0359 (10)	0.0024 (7)	−0.0041 (8)	0.0011 (8)
C4	0.0340 (8)	0.0373 (8)	0.0273 (8)	−0.0021 (6)	0.0038 (8)	−0.0019 (8)
C5	0.0320 (8)	0.0467 (10)	0.0327 (9)	−0.0036 (8)	−0.0052 (8)	−0.0020 (8)
C6	0.0352 (9)	0.0458 (10)	0.0349 (10)	0.0064 (8)	−0.0030 (8)	0.0038 (8)
C7	0.0701 (16)	0.0391 (12)	0.083 (2)	−0.0030 (11)	−0.0173 (15)	0.0059 (12)
C8	0.0468 (11)	0.0391 (11)	0.0599 (16)	−0.0038 (9)	−0.0042 (10)	−0.0035 (9)
C9	0.0571 (12)	0.0463 (11)	0.0654 (15)	−0.0088 (9)	0.0005 (14)	0.0007 (13)
C10	0.0658 (14)	0.0392 (11)	0.080 (2)	0.0088 (10)	0.0024 (14)	0.0063 (12)
C11	0.0502 (12)	0.0381 (10)	0.0655 (15)	0.0066 (9)	0.0077 (11)	0.0001 (10)
Cl1	0.1167 (6)	0.0368 (3)	0.0596 (4)	0.0072 (3)	−0.0038 (4)	−0.0031 (3)

### Geometric parameters (Å, °)

**Table:**

O1—C4	1.378 (2)	C10—C11	1.514 (3)
O1—C7	1.423 (3)	C2—H2	0.9300
N1—C1	1.435 (2)	C3—H3	0.9300
N1—C8	1.463 (3)	C5—H5	0.9300
N1—C11	1.464 (3)	C6—H6	0.9300
N2—C9	1.474 (3)	C7—H7A	0.9600
N2—C10	1.480 (4)	C7—H7B	0.9600
N2—H2A	0.9000	C7—H7C	0.9600
N2—H2B	0.9000	C8—H8A	0.9700
C1—C2	1.393 (3)	C8—H8B	0.9700
C1—C6	1.396 (3)	C9—H9A	0.9700
C2—C3	1.397 (3)	C9—H9B	0.9700
C3—C4	1.379 (3)	C10—H10A	0.9700
C4—C5	1.399 (3)	C10—H10B	0.9700
C5—C6	1.373 (3)	C11—H11A	0.9700
C8—C9	1.520 (3)	C11—H11B	0.9700
			
Cl1···N2	3.134 (3)	H2···H8A	2.2600
Cl1···N2^i^	3.082 (2)	H2···H8B	2.3300
Cl1···H2A^i^	2.2000	H2A···Cl1^vi^	2.2000
Cl1···H9B^ii^	3.1300	H2B···H11A	2.5200
Cl1···H2B	2.2400	H2B···Cl1	2.2400
Cl1···H7A^iii^	2.9700	H2B···H8B	2.5000
O1···H2^iv^	2.7700	H3···C5^iii^	2.8500
O1···H8A^v^	2.6500	H3···C7	2.5100
N1···N2	2.852 (3)	H3···H7B	2.2900
N2···N1	2.852 (3)	H3···H7C	2.2900
N2···Cl1	3.134 (3)	H3···C6^iii^	2.9400
N2···Cl1^vi^	3.082 (2)	H3···C5^vii^	3.0900
N1···H7B^iv^	2.8800	H5···C3^ix^	2.8000
C2···C4^iii^	3.549 (3)	H5···C3^v^	2.9500
C3···C5^vii^	3.435 (3)	H5···C4^ix^	2.8800
C3···C5^iii^	3.585 (3)	H6···C11	2.8700
C3···C4^iii^	3.589 (3)	H6···H11B	2.3000
C4···C2^iv^	3.549 (3)	H7A···Cl1^iv^	2.9700
C4···C3^iv^	3.589 (3)	H7B···C3	2.7400
C5···C3^v^	3.435 (3)	H7B···C1^iii^	2.8700
C5···C3^iv^	3.585 (3)	H7B···H3	2.2900
C1···H7B^iv^	2.8700	H7B···N1^iii^	2.8800
C2···H8A	2.6700	H7B···H8A^iii^	2.5800
C2···H8B	2.8500	H7C···H3	2.2900
C3···H5^viii^	2.8000	H7C···C3	2.7300
C3···H5^vii^	2.9500	H8A···C2	2.6700
C3···H7C	2.7300	H8A···H7B^iv^	2.5800
C3···H7B	2.7400	H8A···O1^vii^	2.6500
C4···H2^iv^	3.0200	H8A···H2	2.2600
C4···H5^viii^	2.8800	H8A···C7^vii^	3.0500
C5···H3^v^	3.0900	H8B···H2B	2.5000
C5···H3^iv^	2.8500	H8B···H11A	2.4600
C6···H11B	2.6800	H8B···C2	2.8500
C6···H3^iv^	2.9400	H8B···H2	2.3300
C7···H8A^v^	3.0500	H9B···Cl1^x^	3.1300
C7···H3	2.5100	H9B···H10A	2.5100
C8···H2	2.5200	H10A···H9B	2.5100
C11···H6	2.8700	H11A···H2B	2.5200
H2···C8	2.5200	H11A···H8B	2.4600
H2···C4^iii^	3.0200	H11B···H6	2.3000
H2···O1^iii^	2.7700	H11B···C6	2.6800
			
C4—O1—C7	116.79 (18)	C6—C5—H5	120.00
C1—N1—C8	115.97 (16)	C1—C6—H6	119.00
C1—N1—C11	114.48 (16)	C5—C6—H6	119.00
C8—N1—C11	110.85 (17)	O1—C7—H7A	109.00
C9—N2—C10	111.0 (2)	O1—C7—H7B	109.00
C9—N2—H2B	109.00	O1—C7—H7C	109.00
C10—N2—H2A	109.00	H7A—C7—H7B	109.00
C10—N2—H2B	109.00	H7A—C7—H7C	109.00
H2A—N2—H2B	108.00	H7B—C7—H7C	109.00
C9—N2—H2A	109.00	N1—C8—H8A	110.00
N1—C1—C2	122.78 (16)	N1—C8—H8B	110.00
N1—C1—C6	119.39 (17)	C9—C8—H8A	110.00
C2—C1—C6	117.76 (16)	C9—C8—H8B	110.00
C1—C2—C3	120.99 (17)	H8A—C8—H8B	108.00
C2—C3—C4	120.16 (18)	N2—C9—H9A	110.00
O1—C4—C5	115.99 (17)	N2—C9—H9B	110.00
O1—C4—C3	124.69 (18)	C8—C9—H9A	110.00
C3—C4—C5	119.31 (18)	C8—C9—H9B	110.00
C4—C5—C6	120.09 (18)	H9A—C9—H9B	108.00
C1—C6—C5	121.68 (18)	N2—C10—H10A	110.00
N1—C8—C9	109.88 (17)	N2—C10—H10B	110.00
N2—C9—C8	109.72 (19)	C11—C10—H10A	110.00
N2—C10—C11	110.0 (2)	C11—C10—H10B	110.00
N1—C11—C10	110.4 (2)	H10A—C10—H10B	108.00
C1—C2—H2	119.00	N1—C11—H11A	110.00
C3—C2—H2	120.00	N1—C11—H11B	110.00
C2—C3—H3	120.00	C10—C11—H11A	110.00
C4—C3—H3	120.00	C10—C11—H11B	110.00
C4—C5—H5	120.00	H11A—C11—H11B	108.00
			
C7—O1—C4—C3	−0.2 (3)	N1—C1—C2—C3	175.8 (2)
C7—O1—C4—C5	−178.8 (2)	C6—C1—C2—C3	−1.2 (3)
C8—N1—C1—C2	−10.2 (3)	N1—C1—C6—C5	−176.6 (2)
C8—N1—C1—C6	166.8 (2)	C2—C1—C6—C5	0.5 (3)
C11—N1—C1—C2	120.9 (2)	C1—C2—C3—C4	1.2 (3)
C11—N1—C1—C6	−62.2 (3)	C2—C3—C4—O1	−179.0 (2)
C1—N1—C8—C9	−168.1 (2)	C2—C3—C4—C5	−0.4 (3)
C11—N1—C8—C9	59.1 (3)	O1—C4—C5—C6	178.4 (2)
C1—N1—C11—C10	167.9 (2)	C3—C4—C5—C6	−0.3 (3)
C8—N1—C11—C10	−58.6 (3)	C4—C5—C6—C1	0.3 (3)
C10—N2—C9—C8	57.6 (3)	N1—C8—C9—N2	−58.2 (3)
C9—N2—C10—C11	−56.9 (3)	N2—C10—C11—N1	56.8 (3)

Symmetry codes: (i) *x*, −*y*, *z*−1/2; (ii) *x*, *y*, *z*−1; (iii) −*x*+1/2, −*y*+1/2, *z*−1/2; (iv) −*x*+1/2, −*y*+1/2, *z*+1/2; (v) *x*+1/2, −*y*+1/2, *z*; (vi) *x*, −*y*, *z*+1/2; (vii) *x*−1/2, −*y*+1/2, *z*; (viii) −*x*+1, *y*, *z*−1/2; (ix) −*x*+1, *y*, *z*+1/2; (x) *x*, *y*, *z*+1.

### Hydrogen-bond geometry (Å, °)

**Table:**

*D*—H···*A*	*D*—H	H···*A*	*D*···*A*	*D*—H···*A*
N2—H2A···Cl1^vi^	0.90	2.20	3.082 (2)	168
N2—H2B···Cl1	0.90	2.24	3.134 (3)	177
C3—H3···CgA^iii^	0.93	2.88	3.573 (2)	133

Symmetry codes: (vi) *x*, −*y*, *z*+1/2; (iii) −*x*+1/2, −*y*+1/2, *z*−1/2.

## References

[bb1] Bruker (2005). *SADABS* Bruker AXS Inc. Madison, Wisconsin, USA.

[bb2] Bruker (2007). *APEX2* and *SAINT* Bruker AXS Inc. Madison, Wisconsin, USA.

[bb3] Farrugia, L. J. (1997). *J. Appl. Cryst.* **30**, 565.

[bb4] Farrugia, L. J. (1999). *J. Appl. Cryst.* **32**, 837–838.

[bb5] Flack, H. D. (1983). *Acta Cryst.* A**39**, 876–881.

[bb6] Lu, Y.-X. (2007). *Acta Cryst.* E**63**, o3611.

[bb7] Sadiq-ur-Rehman, Saeed, S., Ali, S., Shahzadi, S. & Helliwell, M. (2007). *Acta Cryst.* E**63**, o4526.

[bb8] Sheldrick, G. M. (2008). *Acta Cryst.* A**64**, 112–122. 10.1107/S0108767307043930 18156677

[bb9] Spek, A. L. (2009). *Acta Cryst.* D**65**, 148–155. 10.1107/S090744490804362X PMC2631630 19171970

[bb10] Zia-ur-Rahman, Ali, S., Muhammad, N. & Meetsma, A. (2006). *Acta Cryst.* E**62**, m3560–m3561.

[bb11] Zia-ur-Rahman, Ali, S., Muhammed, N. & Meetsma, A. (2007). *Acta Cryst.* E**63**, m89–m90.

